# The complete chloroplast genome sequence of *Liparis yongnoana*, an endemic orchid of Korea

**DOI:** 10.1080/23802359.2019.1644220

**Published:** 2019-07-22

**Authors:** Young-Ho Ha, Hee-Young Gil, Jungsim Lee, Dong Kap Kim, Kyung Choi, Kae Sun Chang, Seung-Hwan Oh

**Affiliations:** a Division of Forest Biodiversity, Korea National Arboretum, Pocheon-si, Republic of Korea;; b Department of Life Science, Gachon University, Seongnam-si, Republic of Korea

**Keywords:** Chloroplast genome, endemic species, Orchidaceae, *Liparis yongnoana*

## Abstract

The complete chloroplast genome sequence of *Liparis yongnoana* was determined and analyzed in this study. The chloroplast genome size is 153,165 bp in length with 36.9% GC content. It comprises a large single-copy region (LSC) of 83,690 bp, a small single copy region (SSC) of 17,661 bp, and a pair of inverted repeat regions (IRa and IRb) of 25,907 bp separated by the SSC. The genome contains 132 genes, including 86 protein-coding, eight ribosomal RNA, and 38 transfer RNA genes. Phylogenetic analysis inferred from 16 Orchidaceae chloroplast genomes suggested that *L. yongnoana* was closely related to *L. loeselii*.

The genus *Liparis* Rich. is a member of tribe Malaxideae within orchid family, Orchidaceae and comprises approximately 320 species occurring in mainly tropical regions and temperate regions of the Northern Hemisphere (Chen et al. 2009). Of six *Liparis* species, *Liparis yongnoana* N.S. Lee, C.S. Lee, & K.S. Lee was recently described as a new species and endemic species of the Korean Peninsula (Chung et al. 2017). This species has similar morphology with *L. japonica* (Miq.) Maxim. and *L. makinoana* Schltr. However, it is distinguished from those species by some morphological characteristics such as fewer number of flowers, more reflexed labellum with a less emarginated apex, and a narrow elliptic line on a labellum (Lee et al. 2010). Although it has been known to be distributed on Jeju Island with a few small populations, several more populations have been found in inland (J. Lee. unpublished). Although phylogenetic position of *L. yongnoana* has been inferred using universal molecular markers and it was monophyletic, the genomic data of *L. yongnoana* have not been explored.

In this study, we characterized the complete chloroplast genome sequence of *L. yongnoana.* The wild individual was collected from Mt. Hambaek, Jeongseon, Gangwon-do, South Korea and a voucher specimen (LJS160003) is deposited in the Herbarium of Korea National Arboretum (KH). Total genomic DNA was extracted from fresh leaves using the DNeasy Plant Mini Kit (Qiagen Inc., Valencia, California, USA). Next-generation sequencing using the Illumina Miseq platform was conducted by Macrogen (Macrogen Inc., Seoul, South Korea). A total of 8,269,920 raw reads were obtained and trimmed (error probability limit: 0.01) using the Geneious R v. 10.2.3 program (Biomatters Ltd., Auckland, New Zealand). The chloroplast genome of *Liparis loeselii* (L.) Rich. (MF374688) was used as a reference genome for assembling the cp genome of *L. yongnoana*. Annotation of the genome was conducted using Dual Organellar GenoMe Annotator (DOGMA) (Wyman et al. 2004) and Geneious Rv.10.2.3 (Biomatters Ltd., Auckland, New Zealand). The tRNAs were confirmed using tRNAscan-SE (Schattner et al. 2005).

The complete chloroplast genome sequence of *L. yongnoana* (GenBank accession MK801140) is 153,165 bp in length with mean coverage depth 113.6×. It showed typical quadripartite structure, in which a large single copy (LSC) region of 83,690 bp, a small single copy (SSC) region of 17,661 bp, and a pair of repeat regions (IRa and IRb) of 25,907 bp. This plastome contained 132 genes, including 86 protein-coding, 38 tRNA, and eight rRNA genes. Among 132 genes, twenty genes (eight protein-coding, eight tRNA, and four rRNA genes) were duplicated in the IR regions. Phylogenetic analysis of subfamily Epidendroideae of Orchidaceae was performed using IQ-TREE v.1.6.8 (Nguyen et al. 2015) based on 67 protein-coding genes with 1000 bootstrap (BS) replications (Figure 1). The ML tree showed that genus *Liparis* is monophyletic and *L. yongnoana* was most closely related to *L. loeselii*.

**Figure 1.**
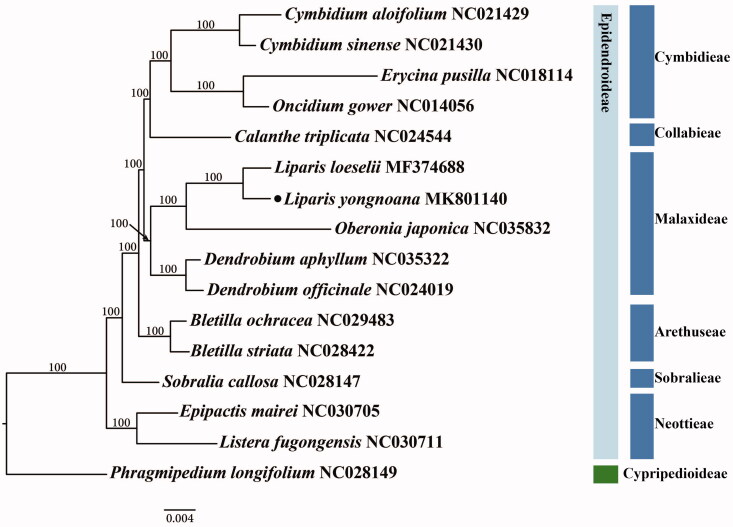
Maximum-likelihood phylogenetic tree of *L. yongnoana* with 15 species belonging to the Orchidaceae based on 67 chloroplast protein-coding sequences. Numbers above the nodes are the bootstrap values from 1000 replicates.
